# Metabolic changes in normal-appearing white matter associate with MRI measures of disease burden in relapsing-remitting multiple sclerosis over three years

**DOI:** 10.1038/s41598-026-45342-2

**Published:** 2026-03-24

**Authors:** Anna Zöchner, Wolfgang Bogner, Assunta Dal-Bianco, Bernhard Strasser, Gilbert Hangel, Paulus Stefan Rommer, Eva Niess

**Affiliations:** 1 High Field MR Center, Department of Biomedical Imaging and Image-Guided Therapy, Medical University of Vienna, Lazarettgasse 14, 1090 Vienna, Austria; 2 Christian Doppler Laboratory for MR Imaging Biomarkers (BIOMAK), Department of Biomedical Imaging and Image-Guided Therapy, Medical University of Vienna, Lazarettgasse 14, 1090 Vienna, Austria; 3 Department of Neurology, Medical University of Vienna, Währinger Gürtel 18-20, 1090 Vienna, Austria; 4 Department of Neurosurgery, Medical University of Vienna, Währinger Straße 18-20, 1090 Vienna, Austria; 5 Comprehensive Center for AI in Medicine (CAIM), Medical University of Vienna, Lazarettgasse 14, 1090 Vienna, Austria

**Keywords:** MRSI, Normal-appearing white matter, Paramagnetic rim lesions, Myo-inositol, N-acetylaspartate, Multiple sclerosis, Biomarkers, Diseases, Neurology, Neuroscience

## Abstract

Myo-inositol (mI) and total N-acetylaspartate (tNAA) are potential MR spectroscopic imaging (MRSI) biomarkers of smouldering-associated worsening in multiple sclerosis (MS). In this study, we explored metabolic changes in normal-appearing white matter (NAWM) in patients with relapsing–remitting MS (pwRRMS) using 7 T MRSI. 20 pwRRMS were scanned annually with 2D-MRSI, with follow-up ranging up to three years. Metabolic ratios were calculated within NAWM for each measurement and assessed cross-sectionally and longitudinally with established and emerging MRI measures of disease burden, including paramagnetic rim lesions (PRLs), brain atrophy, and T2 lesion load. At baseline, pwRRMS showed higher mI/tNAA (β = 0.058; *p* = 0.047) and lower tNAA/total creatine (tCr) (β = − 0.106; *p* = 0.029) compared to controls. tNAA/tCr was further reduced in pwRRMS with PRLs (β = − 0.138; *p* = 0.022). mI/tNAA was associated negatively with cerebral WM volume (β = − 0.001, *p* = 0.015) and positively with T2 lesion volume (β = 0.009; *p* = 0.044). mI/tNAA increased longitudinally in pwRRMS (β = 0.014; *p* = 0.022), especially with higher PRL count (β_timepoint:PRLcount_ = 0.016; *p* = 0.045). An association between tNAA/tCr and the interaction of time with total GM volume was observed but did not survive FDR-correction (β = 0.001; *p* = 0.078). These findings further support the clinical relevance of mI and tNAA alterations as imaging biomarkers of underlying pathology in MS. They highlight the value of 7 T MRSI in capturing subclinical disease burden and underscore the association of PRLs with overall widespread WM damage.

**Supplementary Information:**

The online version contains supplementary material available at 10.1038/s41598-026-45342-2.

## Introduction

Multiple sclerosis (MS) is a chronic autoimmune inflammatory, demyelinating, and neurodegenerative disease of the central nervous system^1^. The most common type of MS is relapsing–remitting MS (RRMS), characterized by alternating periods of acute neurological impairment (relapses) followed by partial or complete recovery (remission)^2^. Conventional MRI is routinely used to diagnose and monitor MS, for example by tracking hyperintense lesions on T2-weighted MRI, but lacks specificity in differentiating inflammation, demyelination, axonal loss and gliosis. Additionally, subtle disease-related changes in the normal-appearing white matter (NAWM) remain undetected on conventional MRI^1,3^. In contrast, magnetic resonance spectroscopic imaging (MRSI) provides metabolic insights into MS pathology, assessing markers for neuroaxonal loss and neuronal dysfunction such as total N-acetylaspartate (tNAA) and astroglial activation such as myo-inositol (mI). Metabolic alterations, particularly of tNAA, may precede brain atrophy, providing a more sensitive and earlier assessment of neurodegenerative activity^4,5^. Elevated mI levels and decreased tNAA in MS have been reported previously^6^. Moreover, an increased mI/tNAA ratio in NAWM has been linked to clinical disability progression^7^, highlighting its potential as a predictive marker since the ratio reflects astrogliosis and axonal damage in WM^8,9^. Gliosis and neuronal damage both play crucial roles in the pathology of MS and are considered major drivers of disease progression even in the absence of clinical relapses—a phenomenon termed progression independent or relapse activity (PIRA). Elucidating its underlying mechanisms and identifying reliable biomarkers remain critical unmet clinical needs^10^.

While direct comparisons between 3 and 7 T MR(S)I remain an active area of research, recent high-field (7 T) MRSI studies have shown increased signal-to-noise (SNR) ratio and spatial resolution, as well as, demonstrated feasibility of acquiring high-resolution maps in vivo, which may improve potential for detecting metabolically abnormal WM and for evaluating disease burden^5,9,11^. Due to technological and time constraints only few longitudinal MRS studies have been conducted so far and mainly focus on differences in metabolic trajectories among patient cohorts (e.g., secondary-progressive vs. relapsing–remitting MS)^12,13^. However, little is known about the relationship between longitudinal metabolic changes and established clinical and MRI measures of disease burden, such as T2 lesion load, brain atrophy, Expanded Disability Status Scale [EDSS], which would further support the clinical relevance of these neurometabolic alterations. Among emerging imaging biomarkers, paramagnetic rim lesions (PRLs), a subtype of chronic active lesions, have gained attention as potential biomarker of chronic neuroinflammation and smouldering-associated worsening in MS^14^. PRLs are best visualized using 7 T susceptibility-sensitive MRI like SWI, where they appear as lesions with a characteristic hypointense rim, reflecting iron-laden, proinflammatory microglia and macrophages^15,16^. PRLs are associated with more severe tissue damage, including pronounced demyelination and axonal degeneration in the periplaque region^15,17,18^ elevated serum neurofilament light chain levels, and reduced retinal layer thickness^18,19^. Clinically, they have also been linked to higher relapse rates and greater disability progression as measured by the EDSS^19,20^ and proposed as a supportive imaging biomarker in the 2024 update of the McDonald diagnostic criteria for MS^21^.

In this prospective, cross-sectional, and longitudinal study we investigated metabolic alterations in regional NAWM of patients with relapsing–remitting MS (pwRRMS) using 7 T 2D-MRSI and their changes over time. Baseline ratios were compared between pwRRMS and healthy controls. Relationship between metabolic changes and clinical as well as established and emerging MRI measures of disease burden were examined at baseline and longitudinally during three-years follow up to further explore the promising role of MRSI for monitoring disease activity and progression in MS.

## Results

### Participants

**Fig. 1:**
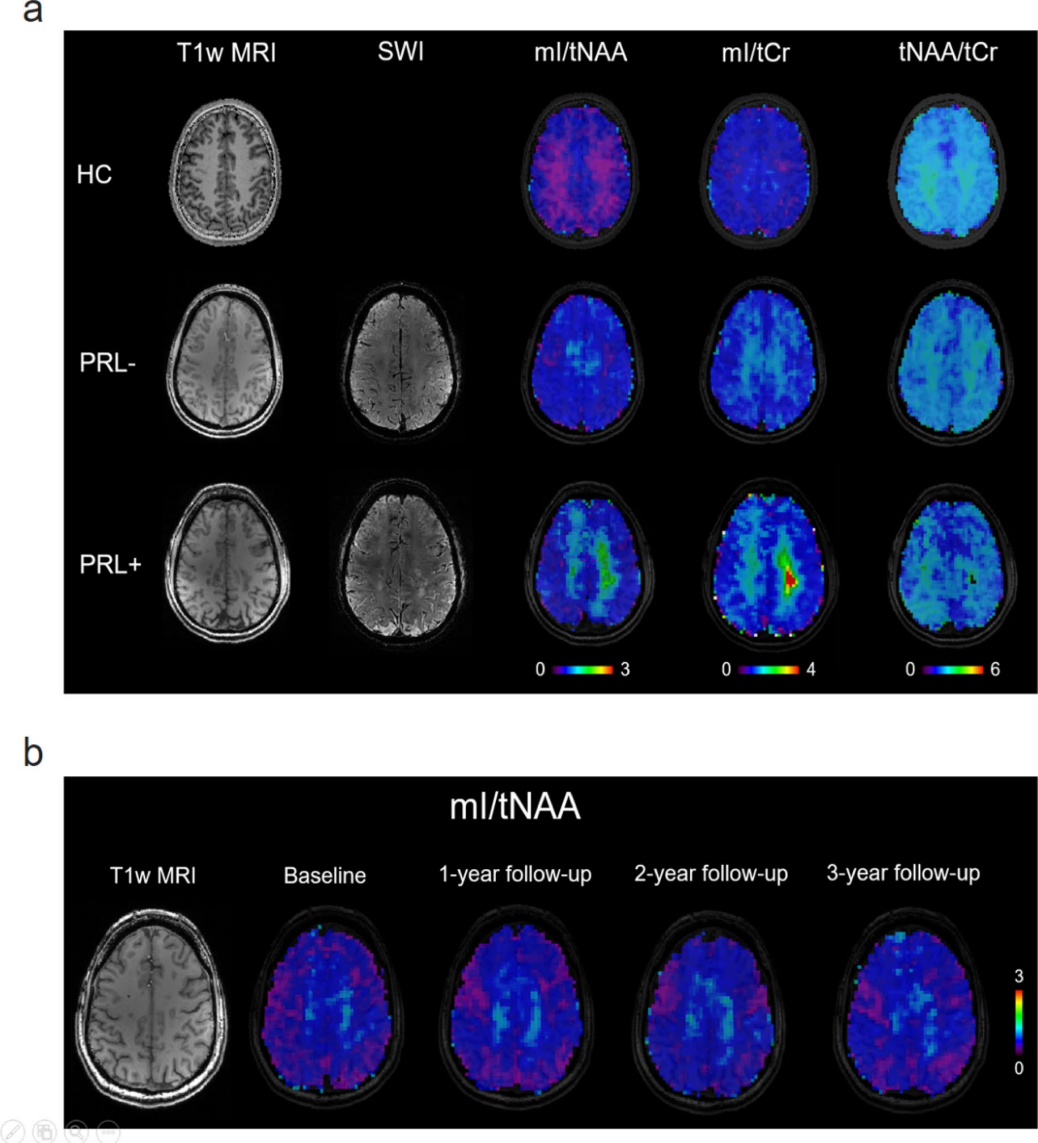
Exemplary structural and metabolic maps in healthy controls and patients with relapsing–remitting multiple sclerosis (pwRRMS). (**a**) Exemplary T1-weighted images and myo-inositol to total N-acetylaspartate (mI/tNAA),  mI to total creatine (mI/tCr) and tNAA/tCr maps of healthy control, pwRRMS with and without paramagnetic rim lesion (PRL + or PRL-, respectively). (**b**) Exemplary mI/tNAA maps of one pwRRMS over all 4 time points.

Example metabolic maps can be found in Fig. 1. The final cohort included 20 patients (median age [range] at baseline, 34 years [25–50]; median EDSS score at baseline [range], 1.25 [0–3]; median disease duration at baseline 8.71 years [0.92–16.7]). Patients were scanned at least twice and up to four times, with a mean follow-up period of 14 months ± 3.5. 85% of patients had at least 3 time points. 100% of patients had at least one follow-up, 85% of patients had at least two-follow ups and 45% had three follow-ups. In total, at baseline 214 lesions were segmented in pwRRMS of which 36 were PRLs, with an average of 2 PRLs per patient. Based on the PRL count 10 pwRRMS were assigned to PRL + and 10 pwRRMS to PRL- subgroups. Overall lesional activity remained largely stable during the study period: only three patients developed new T2 lesions, of whom two had a new PRL. Lesion load was increased at the final time point; however, sample size declined from 20 at baseline to 9 at the final follow-up. The median number of relapses during the observation period was 0 [0–2]. Participant demographics are summarized in Table 1 and in Table 2 explicitly for the PRL subgroups.

**Table 1:** Demographic and clinical data of the study cohort.

Variable	**pwRRMS (n = 20)**	**HC (n = 20)**	***P***
Number of patients (total)	20	20	
Baseline	20	20	
1-year follow-up	18		
2-year follow-up	17		
3-year follow-up	9		
Age in years at baseline; median [range]	33.5 [25–50]	32.5 [24–53]	0.7969
Gender, male; n (%)	13 (65%)	13 (65%)	1
Disease duration in years at baseline; median [range]	8.71 [0.92–16.7]	**–**	
EDSS, median [range]	1 [0–4.5]		
Baseline (n = 20)^a^	1.25 [0–3]		
1-year follow-up (n = 18)^a^	1.25 [0–3]		
2-year follow-up (n = 17)^a^	1.5 [0–4.5]		
3-year follow-up (n = 9)^a^	1 [0–2.5]		
DMT medication, n (%)	17 (85%)	–	
High efficacy	2 (10%)		
Moderate efficacy	3 (15%)		
Low efficacy	12 (60%)		
None	3 (15%)		
Relapse count, median [range]	0 [0–1]	–	
Baseline (n = 20)^a^	0 [0–1]		
1-year follow-up (n = 18)^a^	0 [0–2]		
2-year follow-up (n = 17)^a^	0 [0–1]		
3-year follow-up (n = 9)^a^	0 [0–1]		
T2LV [cm^3^]; median [range]	1.9 [0.28–20.1]	–	
Baseline (n = 20)^a^	2.6 [0.28–20.1]		
1-year follow-up (n = 18)^a^	3.5 [0.42–20.3]		
2-year follow-up (n = 17)^a^	2.1 [0.24–20.1]		
3-year follow-up (n = 9)^a^	11.1 [8.9–23.1]		
PRL count at baseline; median [range]^b^	0.5 [0–10]	–	

DMT, disease-modifying treatment (High efficacy: natalizumab, alemtuzumab, rituximab, Moderate efficacy: fingolimod, Low efficacy: dimethyl fumarate, glatiramer acetate, interferon beta 1a); EDSS, expanded disability status scale; T2LV, T2 lesion volume; PRL, paramagnetic rim lesion; RRMS, relapsing–remitting multiple sclerosis. ^a^Number in brackets indicate the number of patients at each time point. ^b^PRL count was stable over time, only two patients developed a new PRL lesion.

**Table 2:** Demographic and clinical data of the PRL subgroups of pwRRMS (patients with ≥ 1 PRL: PRL + , patients with < 1 PRL: PRL-).

Variable	PRL + (n = 10)	PRL- (n = 10)	*P*
Number of Patients (total)	10	10	
Baseline	10	10	
1-year follow-up	10	8	
2-year follow-up	8	9	
3-year follow-up	5	4	
Age in years at baseline; median [range]	33 [25–50]	33.5 [26–50]	0.649
Gender, male; n (%)	9 (90%)	6 (60%)	0.061
Disease duration in years at baseline; median [range]	5.5 [0.9–12.9]	10.7 [6.4–16.7]	0.015
EDSS, median [range]	1 [0–4.5]	1.5 [0–3]	0.070
Baseline (n = 10/10)^a^	1 [0–3]	1.5 [0–3]	
1-year follow-up (n = 10/8)^a^	1 [0–3]	1.5 [0–3]	
2-year follow-up (n = 8/9)^a^	1 [0–4.5]	1.5 [0–3]	
3-year follow-up (n = 5/4)^a^	1 [0–2.5]	1.75 [0–3]	
DMT medication, n (%)	10 (100%)	7 (70%)	0.149
High efficacy	2 (20%)	0 (10%)	
Moderate efficacy	2 (20%)	1 (10%)	
Low efficacy	6 (60%)	6 (60%)	
None	0 (0%)	3 (30%)	
Relapse count, median [range]	0 [0–1]	0 [0–1]	0.310
Baseline (n = 10/10)^a^	0 [0–1]	0 [0–1]	
1-year follow-up (n = 10/8)^a^	0 [0–2]	0 [0–1]	
2-year follow-up (n = 8/9)^a^	0 [0–1]	0 [0–1]	
3-year follow-up (n = 5/4)^a^	0 [0–1]	0 [0–1]	
T2LV [cm^3^]; median [range]	6.4 [0.9–22.0]	1.7 [0.2–23.1]	0.281
Baseline (n = 10/10)^a^	6.4 [0.9–20.1]	1.7 [0.3–17.2]	
1-year follow-up (n = 10/8)^a^	6.4 [1.3–20.3]	1.7 [0.4–10.5]	
2-year follow-up (n = 8/9)^a^	4.6 [0.9–18.7]	1.3 [0.2–20.1]	
3-year follow-up (n = 5/4)^a^	11.1 [1.4–22.0]	8.0 [0.9–23.1]	
PRL count at baseline; median [range]^b^	0.5 [0–10]	0	

DMT, Disease-modifying treatment (High efficacy: natalizumab, alemtuzumab, rituximab, Moderate efficacy: fingolimod, Low efficacy: dimethyl fumarate, glatiramer acetate, interferon beta 1a); EDSS, Expanded disability status scale; T2LV, T2 lesion volume; PRL, paramagnetic rim lesion; pwRRMS, patients with relapsing–remitting multiple sclerosis.

^a^Numbers in bracket indicate the number of patients at each time point grouped according to the column categories (PRL + /PRL−).

^b^PRL count was stable over time, only two patients developed a new PRL lesion.

### Baseline NAWM metabolic and volumetric changes

#### PwRRMS vs. HCs

**Fig. 2:**
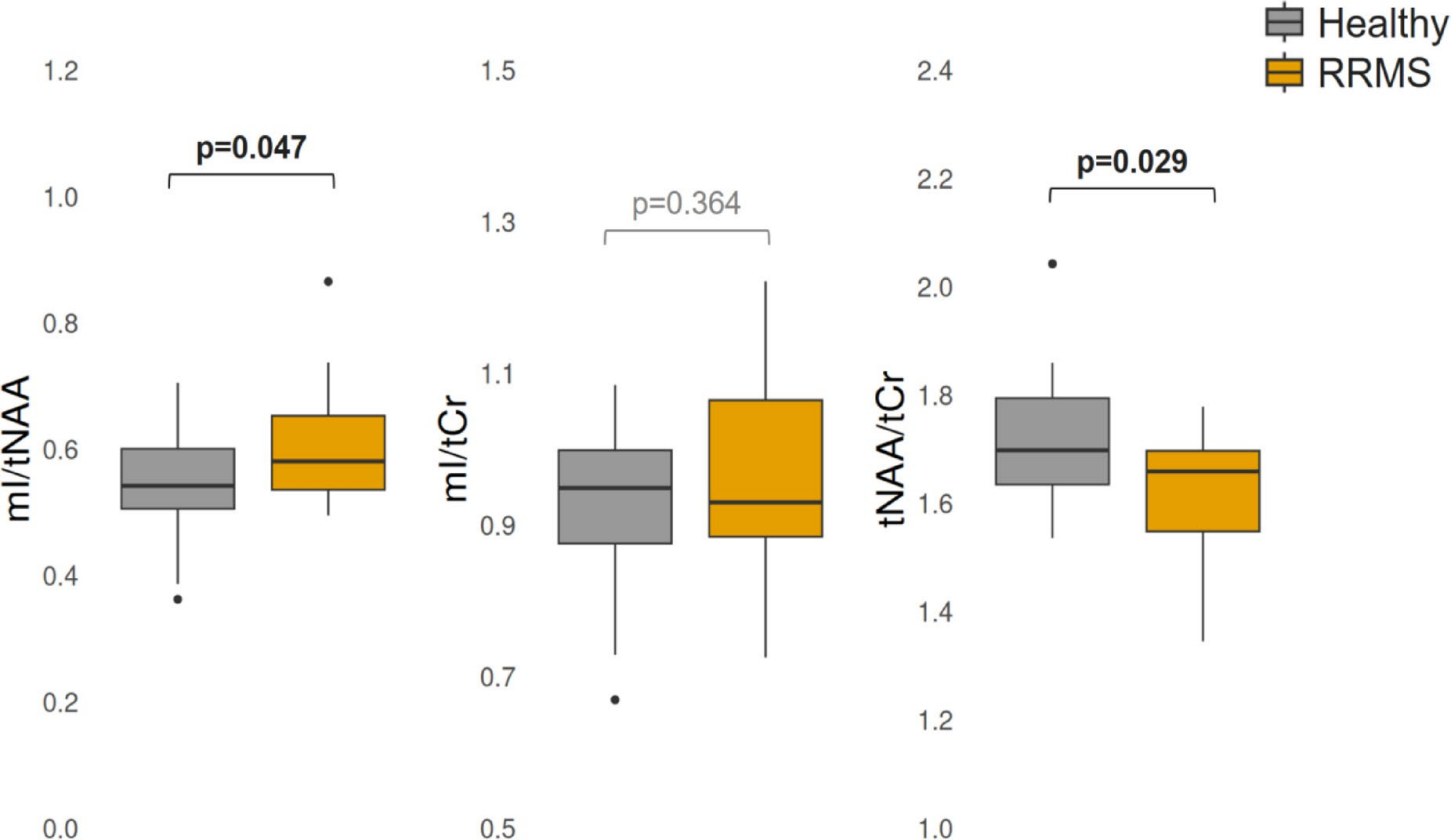
Baseline comparisons of metabolite ratios in patients with relapsing–remitting multiple sclerosis (pwRRMS) and healthy controls. At baseline, myo-inositol to total N-acetylaspartate (mI/tNAA) was higher and tNAA to total creatine (tNAA/tCr) was lower in normal-appearing white matter (NAWM) of pwRRMS compared to healthy controls.

At baseline mI/tNAA was significantly higher (β = 0.058; *p* = 0.047) and tNAA/tCr was significantly lower (β = − 0.106; *p* = 0.029) in pwRRMS compared to HCs (Fig. 2). There were no baseline differences in mI/tCr between the two groups (*p* = 0.364). Results from all linear models with mean values and standard deviation for each group are summarized in Table 3. Volumetric analysis revealed significantly lower GM volume in pwRRMS compared to HCs (β = − 100.307; *p* < 0.001). Mean and standard deviation for each volumetric measure as well as results from linear models can be found in Supplementary Table 1 (S3).

**Table 3:** Results from linear models examining the difference of baseline metabolic ratios in NAWM of HCs and pwRRMS, including mean and standard deviation (SD) for each group.

Metabolite	Mean (SD)	β	95% CI	p-value
mI/tNAA	HCs: 0.55 (0.08)	0.058	0.005–0.110	0.047*
	pwRRMS: 0.60 (0.09)			
mI/tCr	HCs: 0.92 (0.10)	0.031	− 0.038–0.100	0.364
	pwRRMS: 0.96 (0.13)			
tNAA/tCr	HCs: 1.72 (0.12)	− 0.106	− 0.185–(− 0.027)	0.029*
	pwRRMS: 1.61 (0.13)			

SD, standard deviation; β, fixed-effects coefficient; CI, confidence interval; mI, myo-inositol; tCr, total creatine; tNAA, total N-acetylaspartate; HCs, healthy controls; RRMS, relapsing–remitting multiple sclerosis.

Significant values are in bold.

#### PRL subgroups vs. HCs

At baseline, tNAA/tCr was significantly lower (β = − 0.138; *p* = 0.022), while mI/tNAA was higher in PRL + patients compared to HCs (β = 0.068; *p* = 0.065) (Fig. 1a), although the latter did not survive FDR correction. There were no baseline differences observed between the PRL subgroups. Results from all linear models with mean values and standard deviation for each group are summarized in Table 4. Volumetric analysis revealed significantly lower GM volume in PRL + patients compared to HCs (β = − 120.05; *p* < 0.001). The PRL- subgroup also showed lower GM volume compared to HCs but this result did not survive FDR correction (β = − 61.62; *p* = 0.092). Mean and standard deviation for each volumetric measure as well as results from linear models can be found in Supplementary Table 4 (S4).

**Table 4:** Results from linear models examining the difference of metabolic ratios in NAWM of HCs and PRL subgroups, including mean and standard deviation (SD) for each group.

Metabolite	Mean (sd)	Variable	β	95% CI	p-value
mI/tNAA	HC: 0.55 (0.08)				
	PRL− : 0.61 (0.11)	GroupPRL−	0.047	− 0.019–0.113	0.240
	PRL + : 0.60 (0.08)	*GroupPRL* +	0.068	0.002–0.135	*0.065*
mI/tCr	HC: 0.92 (0.10)				
	PRL− : 0.98 (0.12)	GroupPRL−	0.032	− 0.056–0.120	0.468
	PRL + : 0.94 (0.14)	GroupPRL +	0.031	− 0.057–0.118	0.483
tNAA/tCr	HC: 1.72 (0.12)				
	PRL− : 1.63 (0.13)	GroupPRL−	− 0.074	− 0.173–(− 0.025)	0.240
	PRL + : 1.60 (0.13)	**GroupPRL + **	− 0.138	− 0.237–(− 0.039)	0.022*

SD, standard deviation; β, fixed-effects coefficient; CI, confidence interval; mI, myo-inositol; tCr, total creatine; tNAA, total N-acetylaspartate; PRL, paramagnetic rim lesions; HCs, healthy controls; RRMS, relapsing–remitting multiple sclerosis.

Significant values are in bold.

Significant values before FDR correction are in italics.

#### Associations between metabolic ratios and clinical / MRI measures in pwRRMS

Baseline mI/tNAA showed a negative association with cerebral WM volume (β = − 0.001; *p* = 0.015) and a positive association with T2 lesion volume (β = 0.008; *p* = 0.044). In addition, mI/tCr showed a positive association with T2 lesion volume, which did not survive FDR correction (β = 0.008; *p* = 0.057). Results of all linear models at baseline are listed in Table 5.

**Table 5:** Results from linear models examining the association of metabolic ratios in NAWM with clinical and MRI measures at baseline.

Metabolite	Variable	β	95% CI	p-value
mI/tNAA	EDSS	0.042	0.002–0.083	0.112
	Disease duration	0.001	− 0.001–0.002	0.487
	**WM volume [mL]**	− 0.001	− 0.002–0.000	0.015*
	GM volume [mL]	− 0.001	− 0.001–0.000	0.215
	Total intracranial volume [mL]	0.000	0.000–0.000	0.658
	**T2LV [mL]**	0.008	0.002–0.015	0.044*
	PRL count	0.015	− 0.002–0.032	0.260
mI/tCr	EDSS	0.030	− 0.030–0.091	0.306
	Disease duration	0.001	− 0.001–0.003	0.487
	WM volume [mL]	− 0.001	− 0.002–0.000	0.088
	GM volume [mL]	− 0.001	− 0.002–0.001	0.361
	Total intracranial volume [mL]	0.000	0.000–0.000	0.658
	*T2LV [mL]*	0.011	0.002–0.010	*0.057*
	PRL count	0.014	− 0.010–0.039	0.343
tNAA/tCr	EDSS	− 0.053	− 0.019–0.113	0.112
	Disease duration	0.000	− 0.002–0.002	0.934
	WM volume [mL]	0.001	− 0.001–0.002	0.365
	GM volume [mL]	0.001	− 0.001–0.002	0.416
	Total intracranial volume [mL]	0.000	0.000–0.000	0.658
	T2LV [mL]	− 0.003	− 0.008–0.002	0.536
	PRL count	− 0.010	− 0036–0.015	0.403

β, fixed-effects coefficient; CI, confidence interval; mI, myo-inositol; tCr, total creatine; tNAA, total N-acetylaspartate; WM, white matter; GM, gray matter; T2LV, T2 lesion volume; PRL, paramagnetic rim lesion; RRMS, relapsing–remitting multiple sclerosis.

Significant values are in bold.

Significant values before FDR correction are in italics.

### Longitudinal NAWM metabolic and volumetric changes

#### PwRRMS

Over three-years, mI/tNAA increased significantly in pwRRMS (β = 0.014, *p* = 0.022) (see Fig. 1b). A similar upward trend was observed for mI/tCr (β = 0.017, *p* = 0.08). Results from all linear models reflecting the association of metabolites with time are provided in Table 6 with mean values and standard deviations for each time point.

**Table 6:** Results from linear mixed-effects models examining the association of metabolic ratios in NAWM of pwRRMS and time, including mean and standard deviation (SD) for each time point.

Metabolite	Mean (SD)	β	95% CI	p-value
mI/tNAA	Baseline: 0.60 (0.09)	0.014	0.004–0.024	0.022*
	1y follow-up: 0.62 (0.07)			
	2y follow-up: 0.64 (0.09)			
	3y follow-up: 0.63 (0.08)			
mI/tCr	Baseline: 0.96 (0.13)	0.017	0.000–0.033	*0.080*
	1y follow-up: 0.99 (0.1)			
	2y follow-up: 1.00 (0.14)			
	3y follow-up: 0.98 (0.13)			
tNAA/tCr	Baseline: 1.66 (0.13)	− 0.010	− 0.026–0.006	0.219
	1y follow-up: 1.60 (0.11)			
	2y follow-up: 1.59 (0.1)			
	3y follow-up: 1.59 (0.14)			

SD, standard deviation; β, fixed-effects coefficient; CI, confidence interval; mI, myo-inositol; tCr, total creatine; tNAA, total N-acetylaspartate; y, year; RRMS, relapsing–remitting multiple sclerosis.

Significant values are in bold.

Significant values before FDR correction are in italics.

#### Association between longitudinal metabolic changes and clinical / MRI measures in pwRRMS

**Fig. 3:**
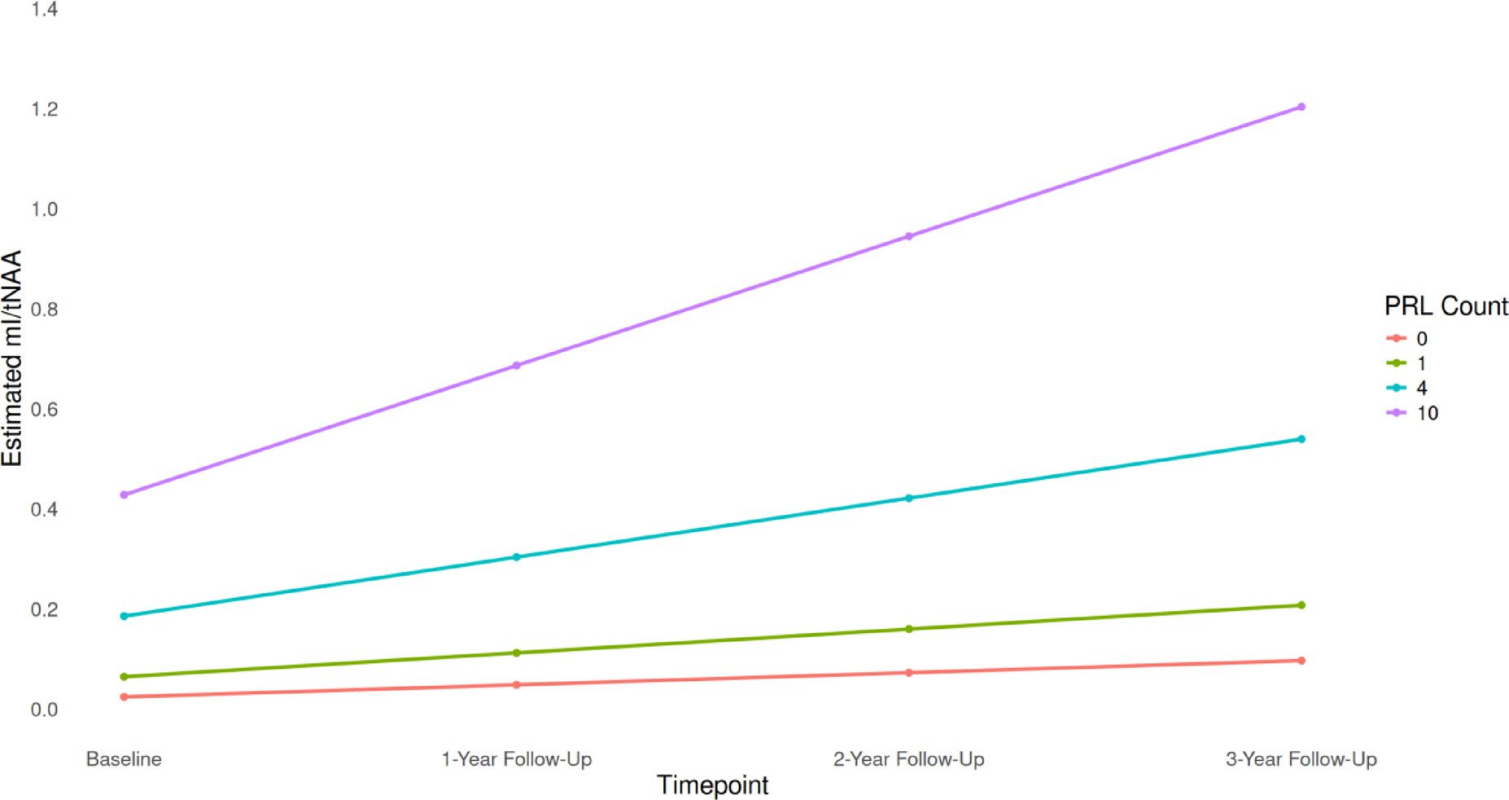
Estimated myo-inositol to total N-acetylaspartate (mI/tNAA) ratio from linear mixed-effects model over time grouped by different paramagnetic rim lesion (PRL) counts. The higher number of PRLs was related to increasing mI/tNAA over time in normal-appearing white matter, supporting the existing evidence of their association with pronounced microstructural damage.

When including all time points in the model, baseline EDSS was significantly positively associated with mI/tNAA (*β* = 0.05, *p* = 0.036) and negatively associated with tNAA/tCr (*β* = − 0.024, *p* = 0.036). No significant associations were observed between any metabolic ratios and the interaction term of time × baseline EDSS. Over the study period no significant changes of WM, GM or total intracranial volume were observed in pwRRMS (Table S5). However, a positive association was found between tNAA/tCr and the interaction of time × total GM volume, but this did not survive FDR correction (*β* = 0.001, *p* = 0.078). A significant positive association was observed between time × PRL count and mI/tNAA (*β* = 0.016, *p* = 0.045) (Fig. 3). In contrast, no association was found between mI/tNAA and time × T2 lesion volume. Results from all linear models regarding longitudinal metabolic changes and clinical / MRI measures in pwRRMS are provided in Table 7.

**Table 7:** Results from each linear mixed-effects model testing for interaction between time and clinical or MRI measure.

Metabolite	Variable	β	95% CI	p-value
mI/tNAA	**Baseline EDSS**	0.05	0.012–0.087	0.036*
	Baseline EDSS:Time	− 0.008	− 0.018–0.000	0.165
	Baseline disease duration:Time	0.000	0.000–0.000	0.707
	WM volume:Time	0.000	0.000–0.000	0.910
	GM volume:Time	0.000	− 0.001–0.000	0.284
	Total intracranial volume:Time	0.000	0.000–0.000	0.789
	T2LV:Time	− 0.001	− 0.002–0.001	0.605
	**PRL count:Time**	0.016	0.004–0.027	0.045*
mI/tCr	Baseline EDSS	0.033	− 0.025–0.091	0.299
	Baseline EDSS:Time	− 0.005	− 0.021–0.011	0.566
	Baseline disease duration:Time	0.000	0.000–0.000	0.707
	WM volume:Time	0.000	− 0.001–0.000	0.969
	GM volume:Time	0.000	− 0.001–0.001	0.788
	Total intracranial volume:Time	0.000	0.000–0.000	0.924
	T2LV:Time	− 0.001	− 0.003–0.001	0.605
	PRL count:Time	0.010	− 0.011–0.032	0.447
tNAA/tCr	**Baseline EDSS**	− 0.066	− 0.118–(− 0.014)	0.036*
	Baseline EDSS:Time	0.013	− 0.002–0.029	0.165
	Baseline disease duration:Time	0.000	0.000–0.000	0.969
	WM volume:Time	0.000	0.000–0.001	0.447
	*GM volume:Time*	0.001	0.000–0.001	*0.078*
	Total intracranial volume:Time	0.000	0.000–0.000	0.726
	T2LV:Time	0.001	− 0.001–0.003	0.605
	PRL count:Time	− 0.009	− 0.030–0.012	0.447

β, fixed-effects coefficient; CI, confidence interval; mI, myo-inositol; tCr, total creatine; tNAA, total N-acetylaspartate; EDSS, expanded disability status score; WM, white matter; GM, gray matter; PRL, paramagnetic rim lesion; RRMS, relapsing–remitting multiple sclerosis.

Significant values are in bold.

Significant values before FDR correction are in italics.

## Discussion

In this prospective cross-sectional and longitudinal 7 T 2D-MRSI study, we examined metabolic alterations in NAWM restricted to the investigated single-slice MRSI region of 20 pwRRMS and 20 controls and explored their relationships with clinical and MRI measures of disease burden. Our key findings demonstrate a progressive increase in mI/tNAA in pwRRMS over a 3-years follow-up, which was significantly associated with the number of PRLs. This indicates that PRLs are linked to pronounced metabolic alterations in NAWM and supports growing evidence that they contribute to widespread WM damage. At baseline, mI/tNAA was associated with T2 lesion volume and cerebral WM volume, while longitudinal tNAA/tCr decline showed a marginal association with total GM volume loss. Together, these results underscore the potential of neurometabolic markers such as mI and tNAA, and particularly their ratio. The ratio was used, as it might serve as a composite marker of both neuroinflammation and neurodegeneration, which both play central roles in MS pathology. It further offers higher sensitivity to early, diffuse and subtle changes in MS, showing better correlation with clinical disability, hence might be useful in longitudinal and treatment studies as a MS-sensitive and potentially prognostic marker. Furthermore, the interpretation of changes in metabolite ratios, was guided by parallel assessment of mI/tNAA, tNAA/tCr and mI/tCr, allowing partial inference regarding the main contributor to each ratio alteration.

At baseline, mI/tNAA was significantly increased and tNAA/tCr significantly decreased in the NAWM of pwRRMS compared to controls. While mI contributes to the ratio, the relative stability of mI/tCr and the drecrease in tNAA/tCr suggest that glial alterations are less prominent in the NAWM of pwRRMS compared to controls. This suggests that these alterations were primarily driven by reduced tNAA levels—indicative of neuroaxonal dysfunction or loss, consistent with MS-related axonal injury, though contributions from glial processes cannot be fully excluded. The reduction in tNAA/tCr was even more pronounced in pwRRMS with PRLs, supporting existing evidence that PRLs are associated with marked axonal damage and chronic active lesion pathology, possibly affecting NAWM focal lesion boundaries^15,18,22^. Although our analysis focused on NAWM rather than solely perilesional tissue, our finding underscores the broader impact of chronic active lesions on surrounding tissue integrity.

We further investigated how longitudinal metabolic changes were associated by T2 lesion volume and PRL count. Notably, we observed a significant positive association between the interaction of PRL count and time and increasing mI/tNAA in NAWM, indicating that higher number of PRLs is linked to more pronounced and progressive WM injury. Previous studies have reported associations between PRLs and global as well as deep gray matter volume loss, clinical disability and worse clinical outcomes^19,23,24^. Our findings provide evidence that PRLs are also associated with pronounced metabolic alterations, highlighting their potential relevance for understanding disease progression and clinical outcomes in patients.

In contrast, T2 lesion volume was positively associated with mI/tNAA in NAWM only at baseline, but no significant longitudinal association was observed. This suggests that although higher T2 lesion burden reflects cumulative tissue damage, it is less sensitive to dynamic, ongoing pathological changes. Metabolic alterations in NAWM appear to be driven primarily by chronically active lesions, rather than by total lesion load, which includes many inactive lesions that may no longer contribute to progressive neurodegeneration^25^.

Over the study period, mI/tNAA increased in the NAWM of pwRRMS, supposedly driven by rising mI levels, as mI/Cr also showed a slight, though non-significant, upward trend (*p* = 0.08). This pattern is indicative of persistent glial activity, consistent with previous findings^13,26^. Longitudinal monitoring of neurometabolites offers a valuable window into the evolving pathology of MS and holds potential as a tool for tracking disease progression. This might be particularly relevant in the context of PIRA, a phenomenon in which disability accrual occurs independent of relapses or new lesional pathology. While traditionally associated with progressive MS forms, growing evidence indicates that PIRA can emerge early in the disease course of RRMS and is linked to poorer long-term outcomes^27^. This highlights the urgent need for sensitive and specific biomarkers capable of detecting subclinical progression. Neurometabolic markers such as mI and tNAA, and their ratio, may help capture subtle neuroinflammatory and neurodegenerative changes, offering the potential for earlier detection of disease activity and more timely, individualized interventions to improve patient outcomes.

At baseline, GM volume was reduced in pwRRMS compared to healthy controls. However, it only reached significance in patients with PRLs. This effect has been reported before especially in newly arising PRLs and together with other brain structure volumes, such as cortex and WM volume^28,29^. We did not observe any significant changes in brain volume over the study period in pwRRMS. Volumetric MRI measures are inherently insensitive to subtle or diffuse tissue changes compared to metabolic imaging and are therefore limited in their ability to detect short-term progression^30^. The absence of detectable volume loss in this study may be further explained by the protective effect of DMTs, which have been shown to reduce brain atrophy^31,32^. Furthermore, it may also reflect the clinical characteristics of our cohort, which consisted of clinically stable patients with minimal to moderate disability (median baseline EDSS = 1.25; baseline EDSS ≤ 3 for all subjects) without significant changes in EDSS scores over the course of our study (median endpoint EDSS = 1). Taken together, these factors underscore the value of more sensitive biomarkers, such as mI/tNAA, which may capture ongoing pathological processes well before structural changes, such as atrophy, become evident.

We observed association between declining tNAA/tCr in NAWM and concurrent GM volume loss over time, though this did not survive FDR correction, suggesting that declining neuroaxonal integrity in the NAWM may parallel or contribute to gray matter atrophy over time. This finding underscores the interconnected nature of MS pathology, where metabolic alterations in NAWM may reflect or even precede more widespread neurodegenerative changes in both GM and WM^33^. The observed negative association between mI/tNAA in NAWM and cerebral WM volume at baseline suggests that microstructural tissue injury corresponds to structural brain loss, supporting the clinical relevance of the observed metabolic alterations.

Previous studies have shown significant correlations between EDSS and metabolite ratios involving mI or tNAA^9,13^. In our cohort, we observed similar trends; however, these associations did not remain significant after correcting for multiple comparisons. This may reflect the influence of statistical adjustments aimed at reducing false positives, as well as limitations of our relatively small cohort (n = 20), which had only minimal to moderate disability. Nonetheless, when including all time points, we found a significant association between higher mI/tNAA and lower tNAA/tCr with baseline EDSS, suggesting that initial clinical status may influence the longitudinal trajectories of these metabolite ratios. Although clinical disability remained largely stable, metabolic alterations may reflect subclinical neuodegenerative or glial changes that are not captured by EDSS. In contrast to prior findings^34^, we did not observe significant associations between metabolite changes and disease duration. This finding is not unexpected, as metabolic alterations in NAWM likely reflect ongoing disease activity such as smouldering inflammation, gliosis, and neurodegeneration, whereas disease duration alone does not capture the heterogeneity in disease course across individuals.

While direct comparison to lower field strengths was not performed in this patient cohort, prior work in healthy controls suggests that 7 T offers higher spectral resolution and SNR, potentially enhancing the detection of subtle metabolic alterations^35^.

Our study has limitations that should be considered when interpreting the results. First, the use of a 2D-MRSI restricts the spatial coverage of the brain regions examined. While this method allows for detailed assessment of a specific area, it may not fully capture the heterogeneity of pathophysiological changes occurring throughout the brain in MS. Accordingly, results should be interpreted as region-specific and cannot be generalized to whole-brain metabolic alterations. A recent 3D-MRSI study demonstrated that metabolic changes predominantly affect motor, cognitive, and sensory brain areas as the disease progresses^6^. However, our study did not examine these regions, as we acquired only a single transverse spectroscopic slab positioned above the ventricles. This region was selected due to its predominant involvement in lesional pathology. Future longitudinal studies should employ newly developed whole-brain 3D-MRSI techniques^36^ to provide a more comprehensive view of the disease’s impact across the entire brain, and well as to enable estimation of metabolite concentrations using water reference scans rather than relying solely on metabolic ratios. This will enable a clearer interpretation of metabolite ratios by investigating changes in individual metabolite concentrations. Healthy controls were only measured at baseline; longitudinal comparisons are limited to within-patient changes for pwRRMS. Follow-up measurements at three years were disrupted by the COVID-19 pandemic, followed by a scanner upgrade which rendered the 2D-MRSI sequence unavailable. As a result, the final time point includes only nine patients, which limits longitudinal analyses but still allows observation of ongoing trends. Due to the small sample size we also did not include DMT type in the linear models, although they may influence inflammatory processes, as inclusion of additional covariates would have increased the risk of overfitting. Accordingly, statistically significant findings—especially those with *p*-values close to conventional significance thresholds—should be considered preliminary. Taken together, these limitations indicate that the results are exploratory and warrant confirmation in larger and independent cohorts.

In conclusion, our findings highlight the value of 7 T MRSI in detecting subtle and progressive metabolic alterations in the NAWM of pwRRMS. The progressive increase in mI/tNAA, particularly in individuals with PRLs, points to widespread WM damage driven by gliosis and loss of neuronal and axonal integrity beyond visible lesion borders, suggesting that metabolic changes in NAWM are primarily driven by chronically active lesions. These results further underscore the potential of PRLs as a specific imaging biomarker of ongoing tissue injury and their relevance to a more severe disease trajectory. Additionally, mI/tNAA may serve as a sensitive marker of subclinical disease progression, offering novel insights into the transition from RRMS to a progressive phase and the heterogeneous clinical dynamics, possibly including PIRA. The clinical relevance of the metabolic changes is reinforced by their associations with MRI and clinical measures of disease burden. Future studies with larger cohorts and longer follow-up are needed to validate these neurometabolic markers and explore their utility in monitoring disease progression and guiding individualized treatment strategies in MS.

## Methods

### Study participants

This study including the experimental protocol was approved by the Ethics Committee of the Medical University of Vienna, Austria (approval number 154/2009). All methods were performed in accordance with relevant guidelines and regulations involving human participants, and in compliance with the Declaration of Helsinki and its later amendments. Written informed consent was obtained from every individual involved. In this prospective study 20 participants were enrolled at the outpatient clinic of Department of Neurology, Medical University of Vienna between May 2016 and April 2020 for annual scanning over four times; however, the number of completed scans per participant varied due to a scanner update and the COVID-19 pandemic. Inclusion criteria were a diagnosis of clinically confirmed MS according to the revised McDonald criteria^37^, age between 18 and 55, and stable treatment or no treatment within the previous six months. The exclusion criteria were contraindication to 7.0-T MRI, presence or history of major psychiatric and neurologic disorders (other than MS), presence of relapse and corticosteroid medication within the previous three months. Additionally, 20 age- and sex-matched cognitively healthy control participants were included. The exclusion criteria for controls were similarly contraindication to 7.0-T MRI and presence or history of major psychiatric and neurologic disorders.

### MRI protocol

All measurements were carried out on a 7.0-T MR Scanner (Magnetom, Siemens Healthineers, Erlangen, Germany) using a 32-channel head coil (Nova Medical, Wilmington, MA, US). The following sequences were obtained at each session: 3D T1-weighted imaging using two magnetization-prepared rapid gradient echoes (MP(2)RAGE) with repetition time (TR) of 5000 ms, echo time (TE) of 4.13 ms, inversion times (TI) of 700 and 2700 ms, 0.75 mm^3^ spatial resolution and acquisition time (TA) of 8:02 min; 3D T2-weighted fluid-attenuated inversion recovery (FLAIR) imaging with TR of 8000 ms, TE of 270 ms, TI of 2180 ms, 0.86 mm^3^ spatial resolution, and TA of 7:14 min; and 3D susceptibility weighted imaging (SWI) with TR of 38 ms, TE of 25 ms, 0.3 × 0.3 × 1.2 mm^3^ spatial resolution and TA of 7:24 min. A transversal, single-slice, two-dimensional free induction decay (FID)-MRSI scan was obtained above the corpus callosum with a flip angle of 45°, an acquisition delay/TR of 1.3/600 ms, a matrix size of 64 × 64, a nominal voxel size of 3.4 × 3.4 × 8 mm^3^, WET water suppression, 1024 spectral points, six-fold parallel imaging acceleration via Controlled Aliasing in Parallel Imaging Results in Higher Acceleration (CAIPIRINHA)^38^ and a scan time of 5:11 min. Reproducible positioning in follow-up scans was achieved with automatic positioning and alignment on the anatomy-related slices using anatomical landmarks (AutoAlign®) and reloading the protocol with saved MRSI position of the previous year. A comprehensive description of the MRSI protocol is provided in Supplementary Table 1 (S1), in accordance with the MRSinMRS (minimum reporting standards for in vivo MRS) consensus guidelines^39^. In addition, mean and standard deviation of CRLB of tCr, tNAA and mI and of SNR and FWHM for each time point can be found in Supplementary Table 2 (S2).

### Data processing

Offline MRSI data processing was performed with an automated in-house developed software written in Matlab (R2017b, MathWorks, Natick, MA, US), including MUSICAL coil combination^40^, 2D-CAIPIRINHA/parallel imaging reconstruction, spatial Hamming filtering, channel-wise noise decorrelation, and lipid signal removal via L2-regularization^41^. Spectral quantification was done in LCModel (version 6.3–1; https://s-provencher.com/lcmodel.shtml) with a simulated basis-set of 17 metabolites and a measured macromolecular baseline^42^ over a spectral evaluation range of 1.8–4.2 ppm. Metabolic images were generated from results of spectral quantification and individually masked based on the metabolite’s Cramér-Rao lower bounds (CRLB < 30%). Although the average CRLBs of the quantified metabolites were typically well below 10% (Table S2), we applied a threshold of < 30% to exclude voxels with poor spectral quality. Only voxels meeting this criterion were included in the analysis of regional metabolic maps. For further analysis, metabolic ratio maps to tCr and tNAA were created.

**Fig. 4:**
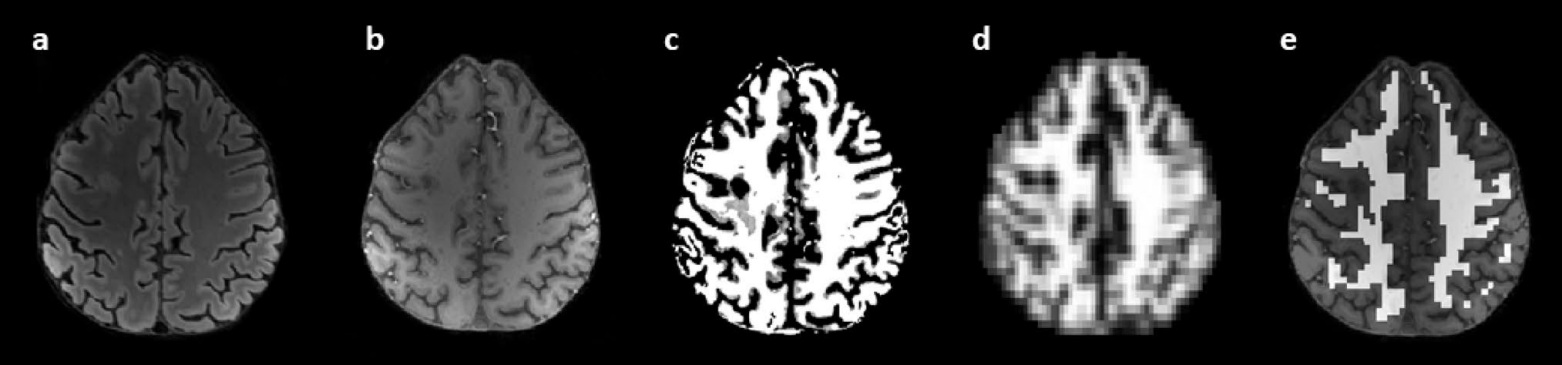
Generation of the normal-appearing white matter (NAWM) mask. (**a**) FLAIR image for anatomical reference. (**b**) T1w image used for tissue segmentation. (**c**) WM probability mask derived from the T1w image using FAST from FSL package. (**d**) WM probability map resampled to the MRSI resolution by downsampling (**c**) in k-space and afterwards converted back to image-space. (**e**) T1w image with the overlaid NAWM mask, which includes only voxels with at least 80% WM content.

T1-weighted images were segmented into WM, GM and cerebrospinal fluid (CSF) probability maps using the FAST tool from the FSL toolbox. To obtain the corresponding tissue volume contributions for each MRSI voxel, the high-spatial resolution anatomical images were Fourier-transformed to k-space and matched to the MRSI resolution before converting the data back to image-space. For each voxel, the fractional WM content was computed and binary white matter masks were generated from voxels containing at least 80% WM content (Fig. 4). Lesion masks were subtracted from the white matter masks to isolate NAWM. For each measurement, metabolite ratios were averaged across all MRSI voxels meeting the WM criterion, resulting in one single WM-representative value per measurement within the investigated MRSI slice. Lesions were manually segmented by an experienced neurologist (A.DB.) on FLAIR-SWI fusion images to assess WML volume and classified as either PRL or non-PRL to obtain PRL count. PRLs were defined as FLAIR-hyperintense lesions that were surrounded by a pronounced and distinct SWI-hypointense rim. For cross-sectional comparisons at baseline, pwRRMS were categorized based on their PRL count into PRL + (pwRRMS with at least one PRL) and PRL- (pwRRMS with no PRL) subgroups. Cerebral WM volume, total GM volume and total intracranial volume were calculated using the FreeSurfer’s recon-all pipeline (version 7.4.1, https://surfer.nmr.mgh.harvard.edu/).

### Statistical analysis

Statistical analysis was conducted in R (version 4.4.1) by a single author (A.Z.). Normality of demographic and clinical data was assessed using the Shapiro–Wilk test. Independent-samples t-tests were applied to normally distributed variables, and Mann–Whitney U tests were used for non-normally distributed variables. Categorical variables were compared using chi-square tests.

Mean metabolic ratios in the NAWM were compared at baseline between healthy controls (HCs) and pwRRMS using linear models. Associations with mean metabolic ratios and clinical and conventional MRI measures were similarly assessed using linear models with the corresponding measure as independent variable. In all models metabolic ratios were analyzed as the dependent variable, adjusted for age and sex. To investigate metabolic and volumetric changes in patient cohort longitudinally, linear mixed-effects models (“lmerTest”, version 3.1.3) were used, with patient ID included as a random effect to account for repeated measurements. Patients with at least two time points were included. Metabolic ratios and volumetric measures were analyzed as the dependent variable, with time, age and sex as fixed effects. In addition, the association between the longitudinal metabolic changes and (1) baseline EDSS, (2) PRL count together with T2 lesion volume and (3) volumetric measures (WM, GM and total intracranial volume) was investigated in the mixed-effects models with an interaction term between the variables of interest and time. DMT type was not included as a variable in the models due to limited sample size and the associated risk of model overfitting. Within each research question *p*-values were adjusted for multiple comparisons using the False Discovery Rate (FDR) method.

Results are reported as estimates (β) with 95% confidence intervals and the corresponding *p*-values, with statistical significance set at *p* < 0.05.

## Supplementary Information

Below is the link to the electronic supplementary material.

Supplementary Material 1

## Data Availability

De-identified participant data can be requested from the corresponding author upon reasonable request with a methodologically sound proposal, subject to participant consent for future data sharing.
