# Supplementary material for: Metabolic changes in normal-appearing white matter associate with MRI measures of disease burden in relapsing-remitting multiple sclerosis over three years
Source: Sci Rep. 2026 Mar 24;16:14808. doi: 10.1038/s41598-026-45342-2 (PMC13168478; doi:10.1038/s41598-026-45342-2)
Supplement: Supplementary file 1 — Supplementary Material 1 [file 41598_2026_45342_MOESM1_ESM.docx]

**SUPPLEMENTARY MATERIAL**

**Table S1:** Minimum Reporting Standards for in vivo MR Spectroscopy (MRSinMRS)

| **Minimum Reporting Standards in MR Spectroscopy checklist (according to Lin et al. NMR Biomed 2021)** | |
| --- | --- |
| **1. Hardware** |  |
| a. Field strength [T] | 7 |
| b. Manufacturer | Siemens |
| c. Model (software version if available) | Magnetom 7T |
| d. RF coils: nuclei (transmit/receive), number of channels, type, body part | 1H, 32 channels, head, Nova Medical |
| e. Additional hardware | N/A |
| **2. Acquisition** |  |
| a. Pulse sequence | FID-MR spectroscopic imaging |
| b. Volume of Interest (VOI) locations | The inferior margin of the slice was positioned at the superior margin of the corpus callosum, angulated to the anterior/posterior commissure line. |
| c. Nominal VOI size [cm³, mm³] | 220 × 220 × 8 mm³ |
| d. Repetition Time (TR), Echo Time (TE) [ms, s] | TR = 600 ms/1.3 ms acquisition delay |
| e. Total number of Excitations or acquisitions per spectrum | 1 average |
| In time series for kinetic studies | N/A |
| i. Number of Averaged spectra (NA) per time-point | N/A |
| ii. Averaging method (eg, block-wise or moving average) | N/A |
| iii. Total number of spectra (acquired/in time-series) | N/A |
| f. Additional sequence parameters (spectral width in Hz, number of spectral points, frequency offsets); If STEAM: Mixing Time TM; If MRSI: 2D or 3D, FOV in all directions, matrix size, acceleration factors | Bandwidth: 6000 Hz, 1024 spectral points, MRSI: 2D, FOV 220 × 220 mm², 8 mm slice thickness, matrix size 64 × 64, Acc. Factor: 6 |
| g. Water Suppression Method | WET |
| h. Shimming Method, reference peak, and thresholds for “acceptance of shim” chosen | Standard shim + manual adjustment, FWHM of the water peak < 40 Hz |
| i. Triggering or motion correction method (respiratory, peripheral, cardiac triggering, incl. device used and delays) | N/A |
| **3. Data analysis methods and outputs** |  |
| a. Analysis software | LCModel 6.3–1 |
| b. Processing steps deviating from quoted reference or prouct | N/A |
| c. Output measure (eg, absolute concentration, institutional units, ratio) | ratio |
| d. Quantification references and assumptions, fitting model assumptions | Simulated in NMRScope-B, macromolecular background |
| **4. Data Quality** |  |
| a. Reported variables (SNR, Linewidth (with reference peaks)) | SNR and FWHM of the LCModel output |
| Outb. Data exclusion criteria | CRLBs > 30% for NAA, tCr, and mI  FWHM>0.1ppm, SNR<5 |
| c. Quality measures of postprocessing Model fitting (eg, CRLB, goodness of fit, SD of residual) | CRLB |
| d. Sample Spectrum See | Supplementary Figure 1 |


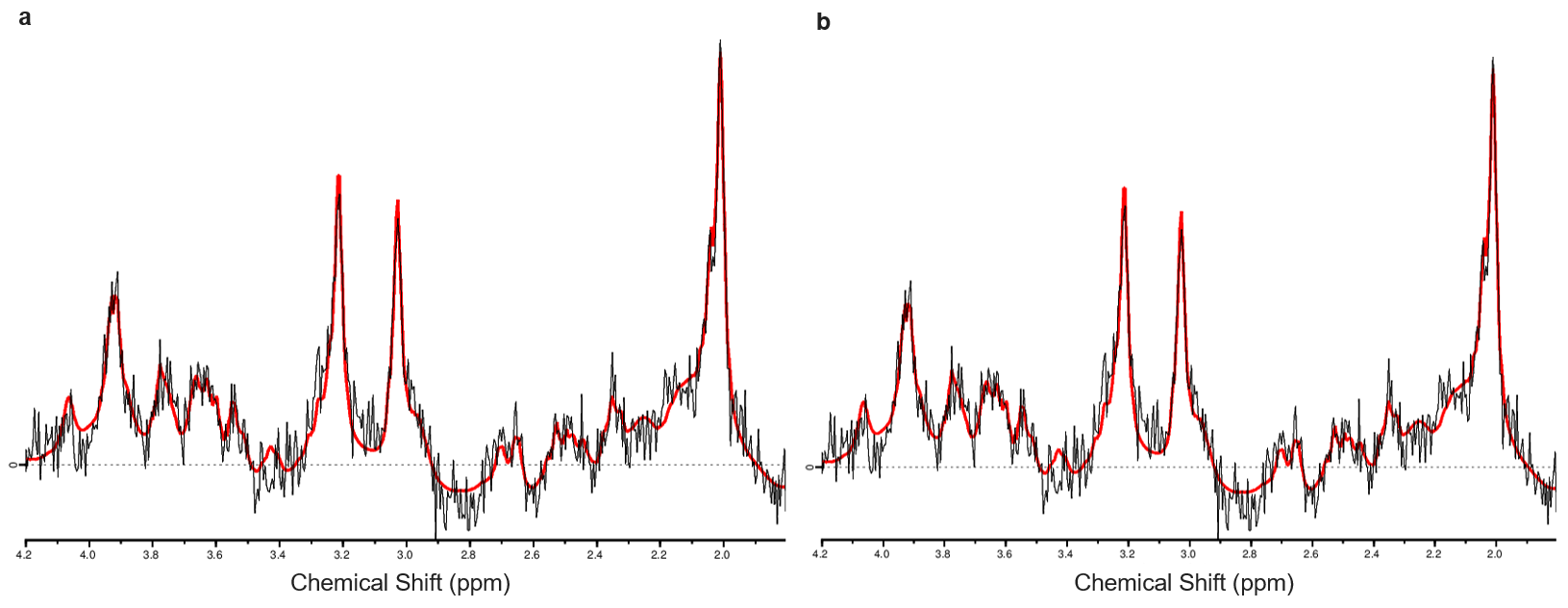


**Supplementary Figure 1:** Exemplary spectrum from NAWM of a patient with RRMS (a) and a healthy control (b).

**Table S2:** Mean and SD values (in brackets) of spectral quality metrics within the NAWM region.

| **Timepoint** | **CRLB mI [%]** | **CRLB tCr [%]** | **CRLB tNAA [%]** | **FWHM [Hz]** | **SNR** |
| --- | --- | --- | --- | --- | --- |
| **Baseline** | 8.65 (1.84) | 6.85 (4.30) | 6.66 (5.28) | 13.17 (2.15) | 7.60 (0.91) |
| **1 year follow-up** | 9.05 (1.78) | 8.14 (5.19) | 9.83 (13.16) | 12.86 (2.59) | 7.34 (1.17) |
| **3 year follow-up** | 9.47 (2.11) | 7.08 (1.87) | 6.56 (3.29) | 13.14 (2.11) | 7.06 (0.95) |
| **3 year follow-up** | 9.67 (3.77) | 6.59 (1.88) | 7.86 (7.43) | 12.07 (1.76) | 7.03 (0.92) |

Note: CRLB = Cramér-Rao Lower Bound, FWHM = Full Width at Half Maximum, SNR = Signal-to-Noise Ratio

**Table S3** Results from linear models examining the difference of volumetric measures in NAWM of HCs and pwRRMS, including mean and standard deviation (SD) for each group.

| **Volumetric Measure** | **Mean (sd)** | **β** | **95 % CI** | **p-value** |
| --- | --- | --- | --- | --- |
| **Cerebral White Matter Volume** | HC: 471 (65.4)  pwRRMS: 466 (58.9) | -4.163 | -40.05-31.72 | 0.815 |
| **Total Gray Matter Volume** | HC: 635 (74.4)  pwRRMS: 533 (53.2) | -100.307 | -139.99-(-60.63) | < 0.001 |
| **Total Intercranial Volume** | HC: 1257 (170)  pwRRMS: 1188 (249) | -59.229 | -196.231-77.773 | 0.579 |

Note: SD = standard deviation; β = fixed-effects coefficient; CI = confidence interval; mI = myo-inositol; tCr = total creatine; tNAA = total N-acetylaspartate; PRL = paramagnetic rim lesions; HCs = healthy controls; RRMS = relapsing-remitting multiple sclerosis

**Table S4** Results from linear models examining the difference of volumetric measures in NAWM of HCs and pwRRMS subgroups, including mean and standard deviation (SD) for each group.

| **Volumetric Measure** | **Mean (sd)** | **β** | **95 % CI** | **p-value** |
| --- | --- | --- | --- | --- |
| **Cerebral White Matter Volume** | HC: 471 (65.4)  PRL-: 462 (54.8)  PRL+: 453 (68.2) | 9.88  -14.87 | -43.19-62.95  -54.73-24.00 | 0.708  0.567 |
| **Total Gray Matter Volume** | HC: 635 (74.4)  PRL-: 549 (63.1)  PRL+: 518 (55.0) | -61.62  -120.05 | -117.12-6.12  -161.74-(-78.36) | 0.051  **< 0.001** |
| **Total Intercranial Volume** | HC: 1257 (170)  PRL-: 1256 (186)  PRL+: 1127 (282) | 45.94  -121.89 | -151.03-242.91  -269.86-26.07 | 0.684  0.155 |

Note: SD = standard deviation; β = fixed-effects coefficient; CI = confidence interval; mI = myo-inositol; tCr = total creatine; tNAA = total N-acetylaspartate; PRL = paramagnetic rim lesions; HCs = healthy controls; RRMS = relapsing-remitting multiple sclerosis

**Table S5:** Results from linear mixed-effects models examining the association of volumetric measures of pwRRMS and time.

| **Volumetric Measure** | **β** | **95 % CI** | **p-value** |
| --- | --- | --- | --- |
| **Cerebral White Matter Volume** | 0.976 | -6.934-8.839 | 0.809 |
| **Total Gray Matter Volume** | 5.265 | -7.417-18.083 | 0.671 |
| **Total Intercranial Volume** | -30.671 | -108.200-49.395 | 0.671 |

Note: β = fixed-effects coefficient for time; CI = confidence interval; RRMS = relapsing-remitting Multiple Sclerosis
